# Prognostic Value of Syndecan-1 in the Prediction of Sepsis-Related Complications and Mortality: A Meta-Analysis

**DOI:** 10.3389/fpubh.2022.870065

**Published:** 2022-04-11

**Authors:** Ting Sun, Yuqiong Wang, Xiaojing Wu, Ying Cai, Tianshu Zhai, Qingyuan Zhan

**Affiliations:** ^1^Capital Medical University China-Japan Friendship School of Clinical Medicine, Beijing, China; ^2^Peking University China-Japan Friendship School of Clinical Medicine, Beijing, China; ^3^Department of Pulmonary and Critical Care Medicine, Center of Respiratory Medicine, National Center for Respiratory Medicine, China-Japan Friendship Hospital, Beijing, China

**Keywords:** syndecan-1, sepsis, septic shock, mortality, acute kidney injury, meta-analysis

## Abstract

**Aim:**

Syndecan-1 (SDC-1) has been shown to have a high predictive value for sepsis development, though uncertainty around these results exists. The aim of this meta-analysis was to assess the prognostic ability of SDC-1 in predicting sepsis-related complications and mortality.

**Methods:**

We searched PubMed, EMBASE, Cochrane Library, and Google Scholar databases from January 01, 1990, to March 17, 2021, to identify eligible studies. The search terms used were “SDC-1,” “sepsis,” “severe sepsis,” and “septic shock,” and a meta-analysis was performed using the RevMan 5.4 software.

**Results:**

Eleven studies with a total of 2,318 enrolled patients were included. SDC-1 concentrations were significantly higher in the composite poor outcome group [standardized mean difference (SMD) = 0.55; 95% CI: 0.38–0.72; *P* < 0.001] as well as in deceased patients (SMD = 0.53; 95% CI: 0.40–0.67; *P* < 0.001), patients with septic shock (SMD = 0.81; 95% CI: 0.36–1.25; *P* < 0.001), and patients with acute kidney injury (SMD = 0.48; 95% CI: 0.33–0.62; *P* < 0.001). Statistical significance was also found in the subgroup analysis when stratified by different sepsis diagnostic criteria.

**Conclusion:**

Baseline SDC-1 levels may be a useful predictor of sepsis-related complications and mortality.

**Systematic Review Registration:**

https://www.crd.york.ac.uk/prospero/display_record.php?ID=CRD42021246344, PROSPERO, identifier: CRD42021246344.

## Introduction

Sepsis is a life-threatening condition characterized by a dysregulated response to infection and is associated with organ dysfunction and high mortality rates (1, 2). Early identification of sepsis patients with a high risk of poor outcomes is vital and can reduce mortality and improve prognosis.

Glycocalyx degradation is a critical driver of organ failure in sepsis due to a combination of pathophysiologic insults (3, 4). It is associated with the development of shock (5, 6), acute kidney injury (AKI) (7), coagulopathy (8), acute respiratory distress syndrome (ARDS)/respiratory failure (9, 10), and mortality (9, 11). Identifying biological markers of glycocalyx degradation may be an essential step in improving outcomes in patients with sepsis.

Syndecan-1 (SDC-1) has been identified as one such biomarker (12, 13), with levels of SDC-1, being elevated in some studies (14, 15). Moreover, multiple studies have shown that SDC-1 levels increased in patients with sepsis, including those with severe sepsis and septic shock. However, only a few studies have demonstrated SDC-1 as a prognostic tool and predictive marker of poor outcomes in patients with sepsis (5, 11, 16, 17). Some previous studies have also included patients with severe sepsis and septic shock. The diagnostic criteria for sepsis have changed three times from 1991 to 2016, which complicates generalization across these studies. Moreover, SDC-1 levels are variable across the longitudinal course of sepsis (11, 18, 19).

The aim of this meta-analysis was to examine the prognostic value of SDC-1 levels upon admission as a predictor of sepsis-related complications and mortality.

## Materials and Methods

This study was conducted in accordance with the Preferred Reporting Items for Systematic Reviews and Meta-Analyses (PRISMA) guidelines. The protocol was registered with PROSPERO (CRD42021246344).

### Search Strategy and Study Selection

A systematic search of the literature across the PubMed, EMBASE, Cochrane Library, and Google Scholar databases from January 01, 1990, to March 17, 2021, was performed using the following keywords: “sepsis,” “severe sepsis,” “septic shock,” and “SDC-1.” We excluded review articles, letters, communications, case reports, and articles published in languages other than English. The reference lists of articles were also reviewed to identify additional relevant studies.

Studies containing the following were included: (1) a prospective study method, (2) patient cohorts aged >18 years, (3) an SDC-1 assessment of serum or plasma within 24 h after admission, and (4) clear diagnostic criteria for sepsis. Moreover, the following reports were excluded: (1) duplicated publications, (2) studies with data not reported or data that could not be transformed into a mean with the standard deviation (SD), and (3) studies which included patients without sepsis. Two investigators (TS and YW) independently extracted studies that complied with the criteria.

### Data Extraction

A standardized form containing first author, year of publication, admission setting, study design, age, sex, number of participants, serum or plasma concentrations, outcomes, and the standards used to define sepsis was recorded. The mean difference and SD were used to pool data, while other forms of data were transformed and described as the mean ± SD (20, 21). For this meta-analysis, “poor outcome” was a composite measure, incorporating mortality and sepsis-associated complications, including septic shock, AKI, disseminated intravascular coagulation (DIC), and ARDS.

Two authors (YW and XW) performed the data extraction independently, using the Newcastle-Ottawa Scale (NOS) to assess the quality of the observational studies. The NOS assigns studies a score of up to nine points based on subject, comparability, and the outcome of interest assessed, with a score of ≥6 indicating a high-quality study.

### Diagnostic Criteria

Sepsis and septic shock definitions were based on three criteria: sepsis 1 (ACCP/SCCM 1991) (22), sepsis 2 (SCCM/ACCP/ATS/SIS 2001) (23), or sepsis 3 (SCCM/ESICM 2016) (24). The diagnosis of AKI was based on either the Acute Kidney Injury Network (AKIN) (25) or Kidney Disease Improving Global Outcomes (KDIGO) (26) criteria. Diagnoses of DIC and ARDS were based on the criteria specified by the International Society of Thrombosis and Hemostasis (27) and Berlin ARDS definition 2012 (28), respectively.

### Statistical Analysis

For this meta-analysis, we used the Review Manager 5.4 (Cochrane Collaboration) software to investigate the association between SDC-1 and poor outcome. Heterogeneity between studies was assessed using the χ^2^ test and inconsistency index (*I*^2^). An *I*^2^ > 50% with *P* < 0.05 was considered indicative of significant heterogeneity. In such cases, a random effect model was chosen, where each measure for poor outcome was then sub-analyzed to explore the source of heterogeneity. Otherwise, a fixed effect model was used. We evaluated publication bias by examining funnel plots when the number of studies reporting the primary clinical outcomes was 10 or more. All tests were two-tailed, and *p* < 0.05 was defined as statistically significant.

## Results

### Results of Literature Search

**Figure 1:**
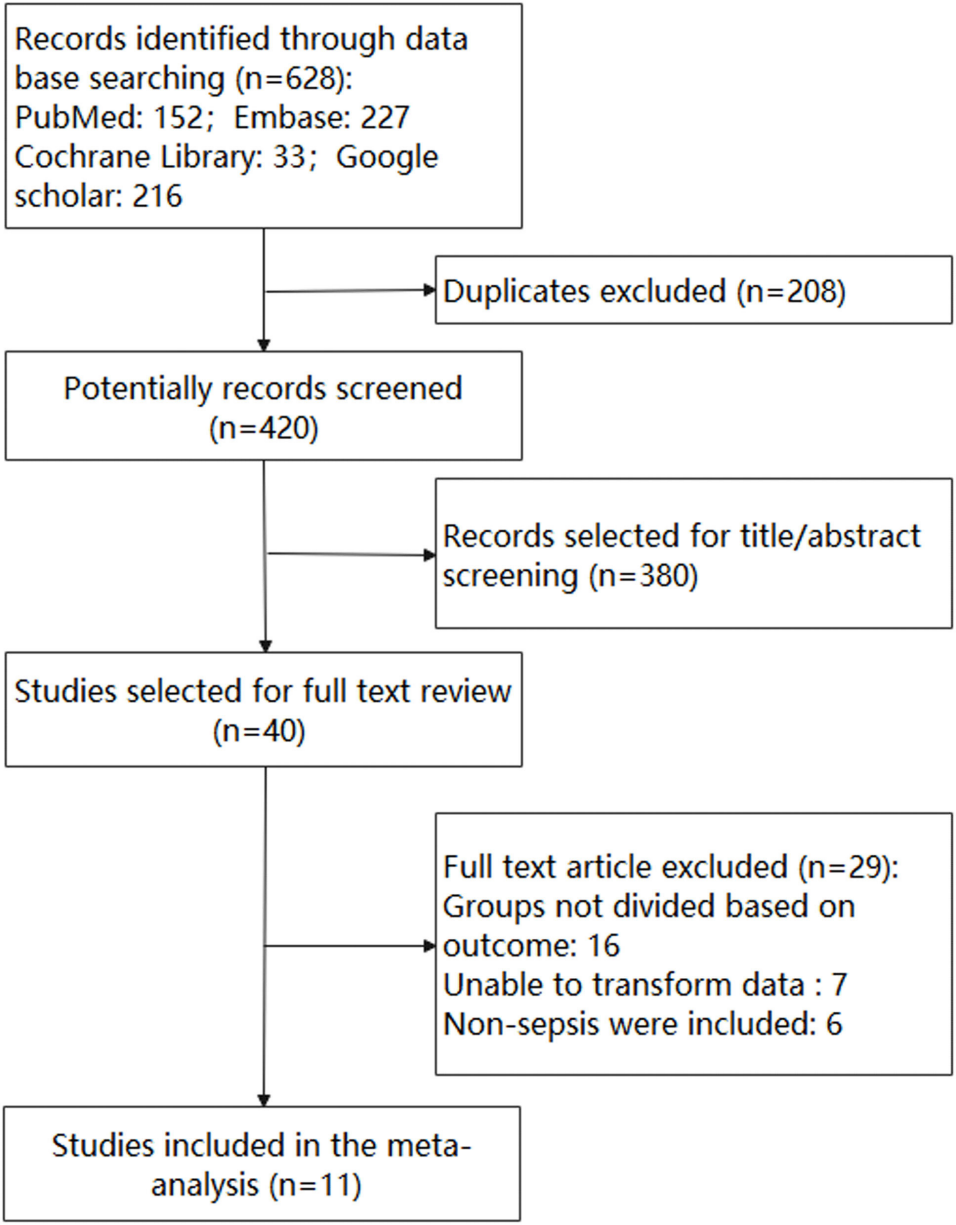
Flow diagram for the identification of eligible studies.

Our initial search of the databases led to the identification of 628 reports, of which 208 were duplicates and subsequently discarded. The titles and abstracts of the remaining 420 reports were then screened, after which, 380 reports were discarded. The full-text articles for 40 studies were read. In total, 11 studies conducted in Asia, Europe, and North America met our inclusion criteria. The procedures used for study selection are described in Figure 1.

### Basic Characteristics of the Included Studies

The eligible studies had a total of 2,318 enrolled patients, 1,375 of whom were male (59.3%). The measures of poor outcome examined included mortality in six studies (11, 29–33), septic shock in five studies (11, 33–36), AKI in three studies (29, 30, 37), and DIC in two studies (36, 38). The study characteristics (i.e., country, year, study design, sepsis definition, age, gender, sampling to analysis, and outcome), Sequential Organ Failure Assessment (SOFA) scores of patients in each study, and NOS scores of the 11 studies (range = 6–7) are shown in Table 1.

**Table 1:** Characteristics of included studies.

**References**	**Country**	**Design**	**Setting**	**Sepsis definition**	**SOFA scores**	**Patient characteristic**	**Age (median)**	**Male (%)**	**Sample**	**Assay**	**Outcome**	**NOS**
Anand et al. (11)	India	PC	ICU	Sepsis-2	6 (4–8)	Sepsis	56	61	Serum	ELISA	Mortality/Septic shock	7
Beurskens et al. (31)	Netherland	PO	ICU	Sepsis-3	8 (7–11)	Sepsis	67	43	Plasma	ELISA	Mortality	6
Huang et al. (36)	China	PO	ICU	Sepsis-3	9.4 ± 3.8	Sepsis	66	82.2	Plasma	ELISA	Septic shock/DIC	6
Ikeda et al. (38)	Japan	PO	ICU	Sepsis-1	9 (5–12)	Sepsis	73	66.7	Serum	ELISA	DIC	7
Inkinen et al. (30)	Finland	PC	ICU	Sepsis-1	8 (6–10)	Sepsis and septic shock	66	64	Plasma	ELISA	Mortality/AKI	7
Johansen et al. (35)	Denmark	PO	ICU	Sepsis-1	Not report	Sepsis	Not report	55.4	Serum	ELISA	Septic shock	7
Johansson et al. (34)	Denmark	PO	ICU	Sepsis-2	5 (5–7)	Severe sepsis	66	59	Serum	ELISA	Septic shock	7
Puskarich et al. (29)	USA	PC	ED	Sepsis-2	7 (4–9)	Severe sepsis	61	53	Plasma	ELISA	Mortality/AKI	7
Saoraya et al. (33)	Thailand	PO	ED	Sepsis-3	4.0 (2.0–6.0)	Sepsis	76	62	Plasma	ELISA	Mortality/Septic shock	7
Sexton et al. (32)	USA	PC	ICU	Sepsis-3	9.12 ± 3.96	Sepsis and septic shouk	52	55	Plasma	ELISA	Mortality	7
Yu et al. (37)	USA	PC	ICU	Sepsis-2	Not report	Severe sepsis	55	51	Plasma	ELISA	AKI	6

*PC, prospective cohort; PO, prospective observational; ICU, intensive care unit; ED, emergency department; SOFA, Sequential organ failure assessment score; DIC, disseminated intravascular coagulation; AKI, acute kidney injury; NOS, Newcastle-Ottawa Scale*.

### Meta-Analysis and Subgroup Analysis

**Figure 2:**
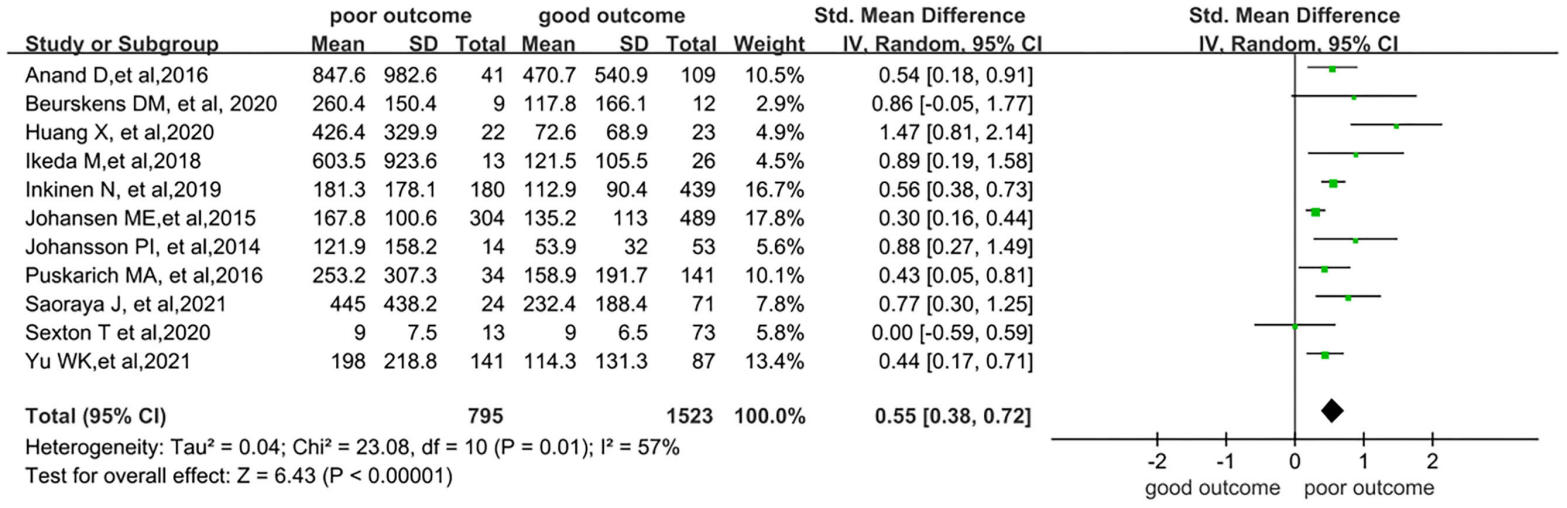
Forest plots of overall poor outcome.

SDC-1 levels were significantly higher in the poor outcome group (standardized mean difference [SMD] 0.55; 95% confidence interval [CI] 0.38–0.72; *I*^2^ = 57%; *p* < 0.001), indicating their potential use for early prediction of poor outcome (Figure 2).

**Figure 3:**
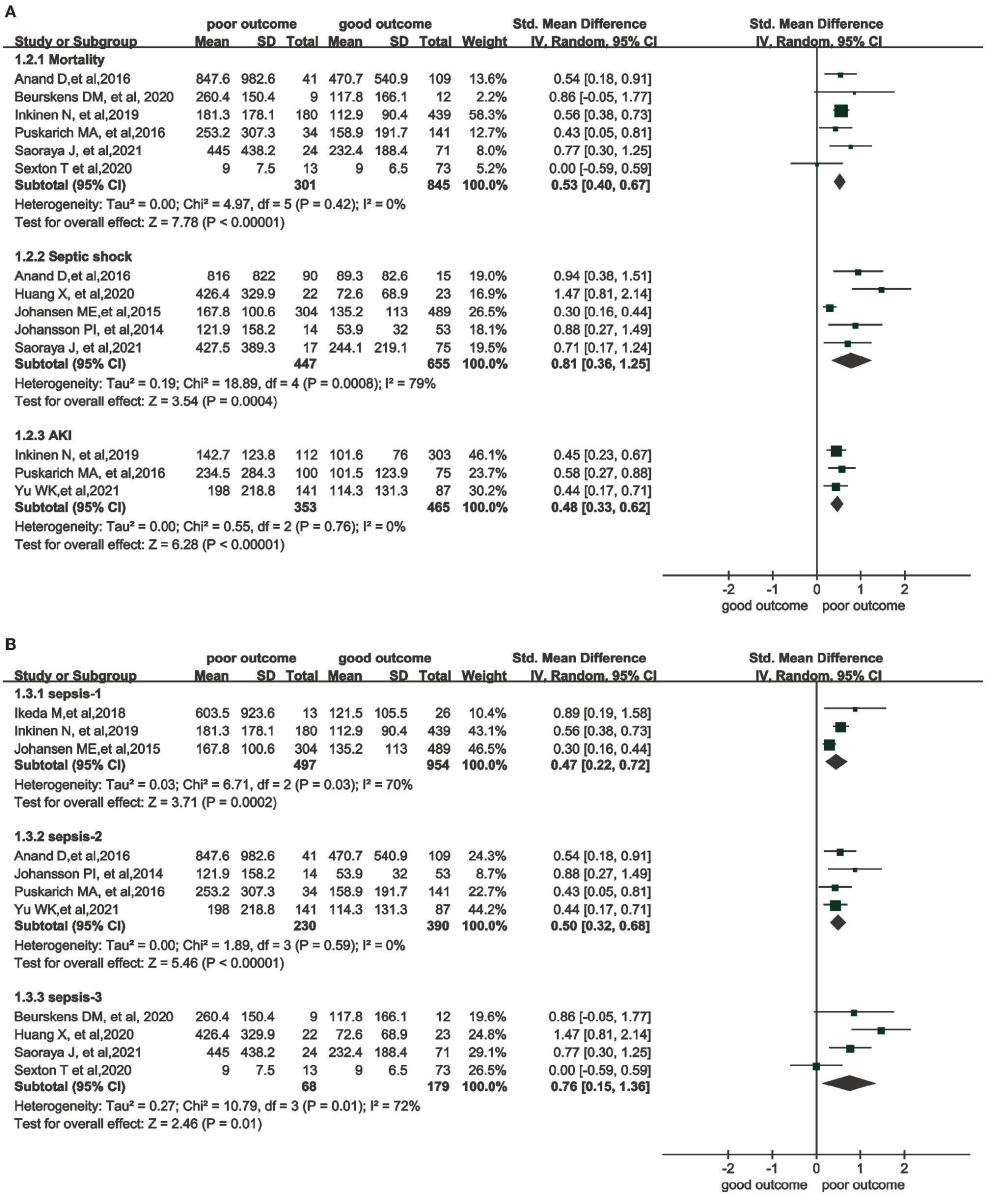
Forest plots of subgroups. **(A)** Subgroup of complications and mortality; **(B)** Subgroup of three diagnostic criterias for sepsis.

Results from the subgroup analysis are presented in Figure 3A. SDC-1 levels were significantly higher in patients who died (SMD = 0.53; 95% CI: 0.40–0.67; *I*^2^ = 0%; *p* < 0.001), as well as in those who developed septic shock (SMD = 0.81; 95% CI: 0.36–1.25; *I*^2^ = 79%; *p* < 0.001) or AKI (SMD = 0.48; 95% CI: 0.33–0.62; *I*^2^ = 0%; *p* < 0.001). Similar results were found in a subgroup analysis when patients were stratified according to the different diagnostic criteria of sepsis 1, sepsis 2, and sepsis 3, as shown in Figure 3B (*p* < 0.001, *p* < 0.001, and *p* = 0.01, respectively). When combining studies which used the same diagnostic criteria, similar results were found.

**Figure 4:**
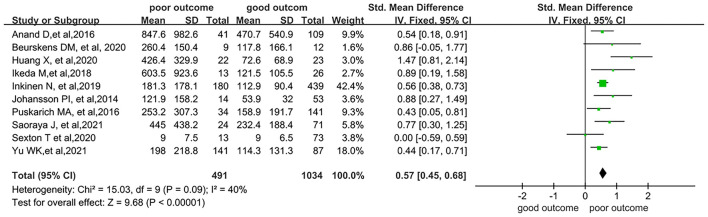
Forest plots of subgroups included 10 studies that meet the sepsis-3 diagnostic criteria.

Among the studies which used the sepsis 1 and 2 diagnostic criteria, five reported SOFA scores (11, 29, 30, 34, 38) ranging from 5 to 9. Another study by Yu et al. (37) only included patients with severe sepsis. According to the sepsis 3 definition (24), patients in these six combined studies, with SOFA scores above 2 or with severe sepsis noted, could be categorized as having sepsis. A meta-analysis including 10 of the studies was also conducted, and a significant difference in SDC-1 levels was noted between patients with poor and good outcomes (SMD = 0.57; 95% CI: 0.45–0.68; *I*^2^ = 40%; *p* < 0.001), as illustrated in Figure 4.

### Sensitivity Analysis

A leave-one-out meta-analysis was performed to detect the influence of heterogeneity on SMD. Sensitivity analysis revealed that heterogeneity decreased when the studies conducted by Huang et al. (from 55 to 36%) and Johansen et al. (from 55 to 40%) were individually removed. When both were removed, heterogeneity was further reduced (from 55 to 0%), and higher SDC-1 levels were noted in the poor outcome group (SMD = 0.54; 95% CI: 0.42–0.66, *p* < 0.001).

### Publication Bias

**Figure 5:**
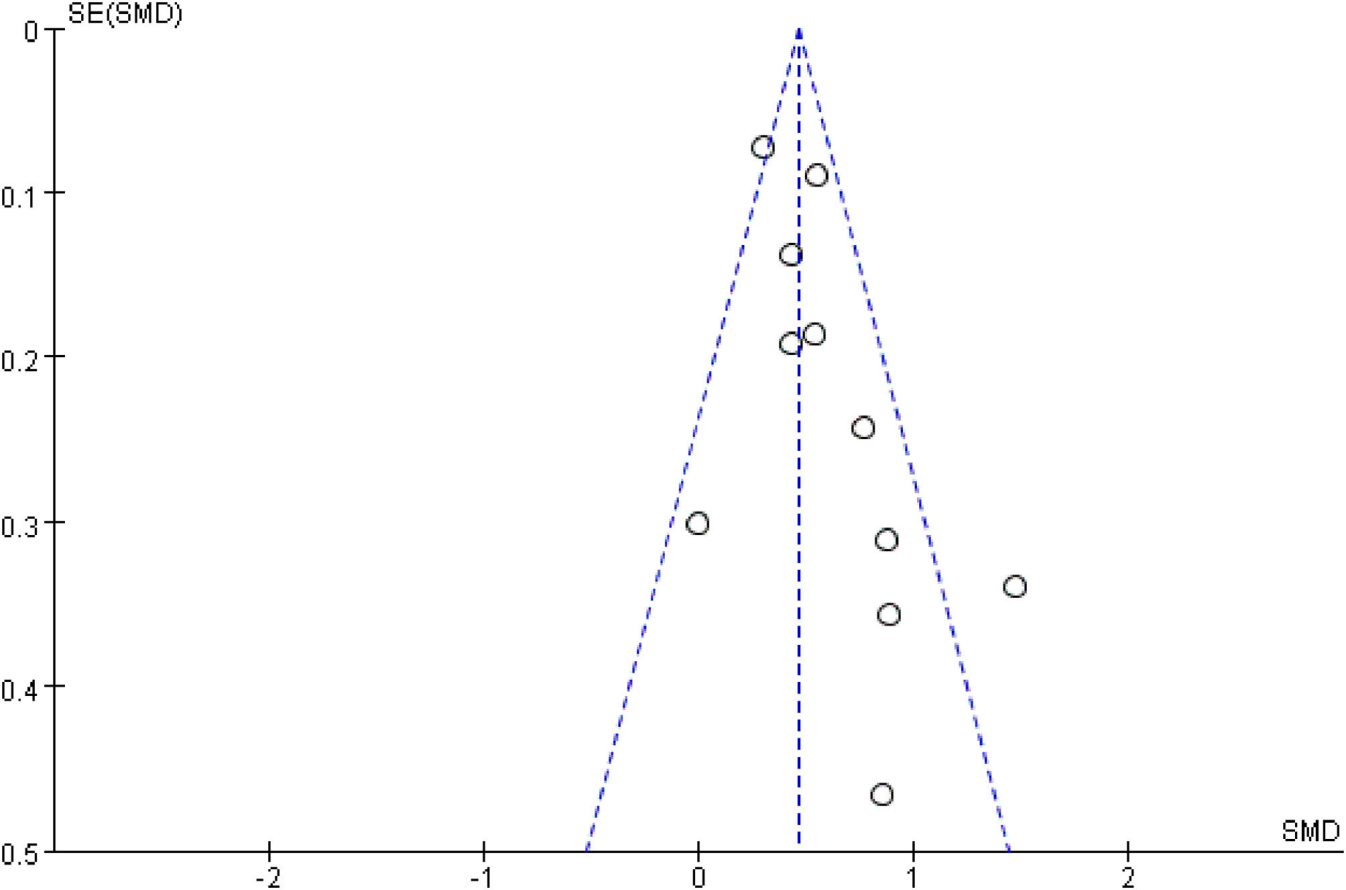
Establish publication bias with funnel plot regarding to all publications.

To evaluate publication bias, the included studies were examined using a funnel plot. A qualitatively symmetrical funnel plot was noted, indicating that no significant publication bias existed in this meta-analysis (Figure 5).

## Discussion

This is the first meta-analysis to examine the prognostic value of baseline SDC-1 levels to predict sepsis-related complications and mortality. SDC-1 levels were higher in the poor outcome group compared with the good outcome group. In a subgroup analysis, SDC-1 levels were significantly higher in deceased patients as well as in those with septic shock or AKI. These results suggest that sepsis patients with higher baseline SDC-1 levels may be at a higher risk of poor outcomes.

A common factor in organ failure is endothelial dysfunction. Degradation of the endothelial glycocalyx occurs in inflammatory states and quickly alters the physiological function of the endothelium, which is implicated in the pathogenesis of critically ill (39), ARDS (40), coronavirus disease 2019 (COVID-19) (41), and pneumonia patients (42). The Sidestream Dark Field (SDF) imaging of the sublingual area is a direct method to assess the thickness of glycocalyx in sepsis patients, but its application is limited by specialized equipment and software. Donati et al. (43) used SDF imaging and found more severe glycocalyx alterations in sepsis patients than in intensive care unit (ICU) patients without sepsis. Beurskens et al. (31) also found significantly lower endothelial glycocalyx thickness in non-survivors than in survivors with sepsis. The most common method for measuring glycocalyx breakdown products is through plasma/serum measurements. SDC-1, a biomarker of glycocalyx degradation, increases with disease severity and is related to poor prognosis in sepsis patients (10, 16, 17, 44). In our meta-analysis, only one of the included studies did not support the prognostic role of SDC-1 in patients with sepsis (32).

However, SDC-1 levels vary over the longitudinal course and relative progression of sepsis (11, 15). Anand et al. (11) found that SDC-1 levels increased over the first week of ICU admission in non-surviving patients with sepsis, compared with those who survived. In the surviving group, SDC-1 levels tended to decrease after the first week. Fraser et al. (45) found a persistent elevation in SDC-1 levels over the first 3 days of ICU admission in patients with COVID-19. In our meta-analysis, the unified selection criteria included prospective studies where SDC-1 levels were measured within the first 24 h after admission, which allowed us to further confirm the prognostic value of SDC-1 for the outcome prediction in patients with sepsis.

Although, there was significant heterogeneity across the 11 studies included in this review, sensitivity analyses indicated that the pooled results were robust. In sensitivity analysis testing, similar results were found when the two studies by Johansen et al. (35) and Huang et al. (36) were removed. The present meta-analysis suggests that SDC-1 may be a useful biological marker for the prediction of sepsis-related complications and mortality.

In the subgroup analysis, we found considerably higher heterogeneity in the septic shock and sepsis 3 subgroups. In the septic shock group, two studies used sepsis 2 criteria, two used sepsis 3 criteria, and only one used sepsis 1 criteria. Therefore, we speculated that the heterogeneity may have been due to the different diagnostic criteria of sepsis, as the diagnosis of septic shock varied considerably across the three criteria. A subgroup analysis, which included 10 studies that all met the sepsis 3 criteria, was also performed. Significantly higher concentrations of SDC-1 were observed in this subgroup compared with patients with good outcomes.

Despite the results of our meta-analysis, the use of a single biomarker to predict sepsis may not always be reliable. We hope that ongoing randomized trials (NCT 04718623 and NCT 04644302) will include a more in-depth analysis of the predictive markers for patients with sepsis.

This meta-analysis had several limitations. First, SDC-1 levels had a high SD, indicating a high level of variability. SDC-1 levels were reported using medians and interquartile range, which were then used to calculate the means and SDs in this meta-analysis. Second, the sample sizes of the included publications were small. Although we pooled the results of these publications, it may still have been possible to miss the effectiveness of the meta-analysis. Third, the included studies used different definitions of sepsis, which may have affected our results. In particular, the definition of septic shock was different, which could partially explain the substantial heterogeneity noted in the septic shock subgroup. However, subgroup and sensitivity analyses indicated that the pooled results were robust. Finally, prospective cohort trials were most qualified for our study objective, as the intervention could not be randomized. Therefore, our meta-analysis of the observational studies, and not of randomized control trials, could only support the potential association between increased SDC-1 and poor outcome in patients with sepsis.

## Conclusion

This meta-analysis supported the prognostic value of SDC-1 as a predictor of mortality and sepsis-related complications.

## Data Availability Statement

The original contributions presented in the study are included in the article/Supplementary Material, further inquiries can be directed to the corresponding author/s.

## Author Contributions

TS and YW extracted studies from the eligible papers. YW and XW performed the data extraction. TZ and YC analyzed the data. TS and QZ wrote the paper. QZ reviewed and revised the paper. All authors contributed to the conception and design of the work.

## Funding

This work was funded by National Natural Science Foundation of China (NO. 81870072); Horizontal subject (NO. 2019-HX-77).

## Conflict of Interest

The authors declare that the research was conducted in the absence of any commercial or financial relationships that could be construed as a potential conflict of interest.

## Publisher's Note

All claims expressed in this article are solely those of the authors and do not necessarily represent those of their affiliated organizations, or those of the publisher, the editors and the reviewers. Any product that may be evaluated in this article, or claim that may be made by its manufacturer, is not guaranteed or endorsed by the publisher.

## Supplementary Material

The Supplementary Material for this article can be found online a: https://www.frontiersin.org/articles/10.3389/fpubh.2022.870065/full#supplementary-material
